# Multidisciplinary Care Approach to Asymptomatic Brugada Syndrome in Pregnancy: A Case Report

**DOI:** 10.3390/reports8030138

**Published:** 2025-08-05

**Authors:** Isabella Marechal-Ross, Kathryn Austin

**Affiliations:** Department of Obstetrics & Gynaecology, Royal North Shore Hospital, Clinical Services Building, 1 Westbourne St, St Leonards, NSW 2065, Australia; kathryn.austin@health.nsw.gov.au

**Keywords:** Brugada syndrome, arrhythmia, channelopathy, pregnancy, obstetrics, antenatal, postpartum

## Abstract

**Background and Clinical Significance:** Brugada syndrome (BrS) is a rare inherited cardiac channelopathy, often associated with SCN5A loss-of-function mutations. Clinical presentations range from asymptomatic to malignant arrhythmias and sudden cardiac death. Physiological and pharmacological stressors affecting sodium channel function—such as pyrexia, certain medications, and possibly pregnancy—may unmask or exacerbate arrhythmic risk. However, there is limited information regarding pregnancy and obstetric outcomes. Obstetric management remains largely informed by isolated case reports and small case series. A literature review was conducted using OVID Medline and Embase, identifying case reports, case series, and one retrospective cohort study reporting clinical presentation, obstetric management, and outcomes in maternal BrS. A case is presented detailing coordinated multidisciplinary input, antenatal surveillance, and intrapartum and postpartum care to contribute to the growing evidence base guiding obstetric care in this complex setting. **Case Presentation:** A 30-year-old G2P0 woman with asymptomatic BrS (SCN5A-positive) was referred at 31 + 5 weeks’ gestation for multidisciplinary antenatal care. Regular review and collaborative planning involving cardiology, anaesthetics, maternal–fetal medicine, and obstetrics guided a plan for vaginal delivery with continuous cardiac and fetal monitoring. At 38 + 0 weeks, the woman presented with spontaneous rupture of membranes and underwent induction of labour. A normal vaginal delivery was achieved without arrhythmic events. Epidural block with ropivacaine and local anaesthesia with lignocaine were well tolerated, and 24 h postpartum monitoring revealed no abnormalities. **Conclusions:** This case adds to the limited but growing literature suggesting that with individualised planning and multidisciplinary care, pregnancies in women with BrS can proceed safely and without complication. Ongoing case reporting is essential to inform future guidelines and optimise maternal and fetal outcomes.

## 1. Introduction and Clinical Significance

Brugada syndrome (BrS) is an autosomal dominant arrhythmic disorder with variable penetrance which affects cardiac ion channels [1,2]. Its clinical presentation is highly variable; while most individuals remain asymptomatic, some experience syncope, palpitations, or chest discomfort. BrS can lead to tachyarrhythmias such as polymorphic ventricular tachycardia (PVT) or ventricular fibrillation (VF), and accounts for almost one-fifth of sudden cardiac deaths (SCD) in patients without structural heart disease [2,3].

BrS is characterised by distinctive electrocardiographic (ECG) features, including right bundle branch block, a normal QT interval, and ST elevation in the right precordial leads, in the absence of myocardial ischaemia or structural abnormality [1,2,3]. Multiple genetic mutations have been implicated, with SCN5A loss-of-function variants being the most common, present in approximately 20% of cases [3,4].

Although BrS is rare, with an estimated prevalence of 0.05 per 1000 individuals worldwide, certain physiological and pharmacological stressors—such as pregnancy, fever, and medications which block the sodium channel—can precipitate malignant arrhythmias [3,5]. The only strategies to prevent SCD in these patients is the avoidance of triggers or implantation of a cardioverter-defibrillator (ICD) [5,6]. As such, particular care must be taken in the obstetric management of affected individuals, given the increased exposure to potential triggers during pregnancy and the postpartum period.

The literature on BrS in obstetric cases remains limited, comprising mostly individual case reports, small case series, and one retrospective cohort study by Rodríguez-Mañero et al. which described the clinical trajectory of 104 women with BrS through a total of 219 pregnancies [7,8]. Of these, only six women experienced a syncopal event, two reported episodes of palpitations, and there were no arrhythmias or SCDs [1,7]. Similar outcomes were reported in a case series by Gualtieri et al. involving 11 obstetric patients with confirmed or suspected BrS; two experienced palpitations and episodes of syncope, while the remaining patients were asymptomatic [9]. No arrhythmic complications occurred during spontaneous vaginal deliveries or caesarean sections [9,10,11]. Importantly, the reported rates of syncope and palpitations were consistent with those documented in the general obstetric population (i.e., without maternal BrS) [1].

Available evidence thus suggests that women with BrS in pregnancy have an overall low risk of developing arrhythmia or SCD [7,8,9,12]. However, the paucity of data poses a significant challenge in determining the clinical implications of BrS in pregnancy, with scarce evidence to inform management strategies or predict perinatal outcomes. This case, managed through a multidisciplinary team approach, is presented to contribute to the growing body of knowledge in this area.

## 2. Case Presentation

A 30-year-old woman, G2P0, was referred to a tertiary centre at 31 + 5 weeks’ gestation for multidisciplinary management of pregnancy in the context of BrS. She had been diagnosed many years earlier via a positive flecainide challenge, following her mother’s diagnosis, which was made in the context of palpitations without syncope. Genetic testing revealed a SCN5A mutation (the most common variant) [2]. The woman was asymptomatic and had previously undergone loop recorder surveillance for three years without arrhythmia detection. Her medical history included L4/L5 disc replacement and scoliosis. She took no regular medications.

Her obstetric history included a spontaneous miscarriage two years prior to this pregnancy. Antenatal screening, including non-invasive prenatal testing (NIPT), was low risk. A morphology scan at 20 weeks’ gestation was unremarkable, and serial growth and wellbeing ultrasounds confirmed appropriate fetal growth, with normal Doppler and amniotic fluid parameters throughout the pregnancy.

Antenatally, the woman was reviewed by her cardiologist. Her baseline ECG was unremarkable. Transthoracic echocardiography (TTE) demonstrated sinus bradycardia (50 bpm), left ventricular systolic function at the lower range of normal with an estimated ejection fraction of 55%, and a minimal posterior pericardial effusion (<1 mm), likely physiological.

The patient had regular antenatal follow up in a specialised multidisciplinary obstetric clinic, with coordinated input from cardiology, anaesthetics, maternal fetal medicine (MFM), obstetrics, and midwifery. She was assessed as suitable for vaginal delivery. Plans were initiated for intra-partum continuous maternal cardiac and fetal monitoring with maternal 3-lead ECG monitoring and continuous electronic fetal monitoring once assessed to be in established labour. Management included strict avoidance of factors known to affect sodium channel function, such as pyrexia, and a comprehensive list of BrS contraindicated medications documented in her electronic medical record [13]. Access details for the BrugadaDrugs.org registry were also included to ensure treating teams had a reliable, evidence-based, and routinely updated resource throughout the peripartum period [13]. Contingency plans were established for post-dates induction of labour and emergency caesarean section, if clinically indicated.

At 38 + 0 weeks, the patient presented with spontaneous rupture of membranes (SROM). Cardiology and anaesthetic teams were notified as per her complex care plan. A large-bore intravenous cannula was inserted, and baseline biochemistry collected, which was normal. Given prolonged SROM without labour onset, intravenous ampicillin was commenced at 18 h, and induction of labour proceeded the following day.

Intrapartum management included early epidural analgesia with 20 mls 0.2% ropivacaine, continuous electronic fetal monitoring, and maternal cardiac monitoring via three-lead ECG with emphasis on V1–V2.

A healthy female infant was born by normal vaginal delivery. Ergometrine and systemic lignocaine were avoided due to their sodium channel-blocking effects, though a second-degree perineal tear was repaired using 5 mL of local lignocaine under senior anaesthetic guidance, without issue. The infant’s BrS genetic variant status is unknown, as the woman declined antenatal genetic testing. The patient remained stable postpartum and underwent 24 h of continuous cardiac monitoring; no arrhythmias were observed, and she was discharged home.

## 3. Discussion

BrS is a rare inherited cardiac channelopathy associated with an increased risk of ventricular arrhythmias and SCD. Its management during pregnancy remains a clinical challenge due to limited data, absence of obstetric-specific guidelines, and the potential for physiological changes and commonly used medications to increase arrhythmic risk.

Most available evidence is drawn from single case reports, small case series, and a single retrospective cohort study. These emphasise the wide phenotypic variability of BrS—from asymptomatic carriers to individuals experiencing life-threatening events. The most common pathogenic SCN5A mutation has been associated with more severe disease and warrants early identification for maternal (and fetal) risk stratification.

In this case of BrS in pregnancy, the patient had an SCN5A variant and diagnostic flecainide challenge, but was asymptomatic and remained so antenatally, intrapartum, and postpartum. Her clinical course was uncomplicated, with no arrhythmic events. Local anaesthesia with lignocaine was well tolerated, and no other contraindicated medications were required. A multidisciplinary approach involving cardiology, anaesthetics, MFM, obstetrics, and midwifery was crucial to providing safe care, with comprehensive antenatal counselling, careful medication planning, and continuous monitoring throughout the intrapartum and postpartum periods.

## 4. Conclusions

This case contributes to the growing body of evidence suggesting that, with individualised planning and close surveillance, pregnancies affected by maternal BrS in the asymptomatic population can be managed without complication. As formal guidelines remain lacking, further case reporting will be critical to guide future management and improve outcomes in this complex clinical context.

## Data Availability

The original contributions presented in this study are included in the article. Further inquiries can be directed to the corresponding author.

## Abbreviations

The following abbreviations are used in this manuscript:

BrS	Brugada Syndrome
PVT	Polymorphic Ventricular Tachycardia
VF	Ventricular Fibrillation
SCD	Sudden Cardiac Deaths
ECG	Electrocardiographic
ICD	Implantable Cardioverter-Defibrillator
NIPT	Non-Invasive Prenatal Testing
TTE	Transthoracic Echocardiography
MFM	Maternal Fetal Medicine
SROM	Spontaneous Rupture of Membranes
